# Recent and Widespread Rapid Morphological Change in Rodents

**DOI:** 10.1371/journal.pone.0006452

**Published:** 2009-07-31

**Authors:** Oliver R. W. Pergams, Joshua J. Lawler

**Affiliations:** 1 Department of Biological Sciences, University of Illinois at Chicago, Chicago, Illinois, United States of America; 2 Division of Mammals, Department of Zoology, Field Museum, Chicago, Illinois, United States of America; 3 Red Rock Institute, Bryn Mawr, Pennsylvania, United States of America; 4 School of Forest Resources, University of Washington, Seattle, Washington, United States of America; Lund University, Sweden

## Abstract

In general, rapid morphological change in mammals has been infrequently documented. Examples that do exist are almost exclusively of rodents on islands. Such changes are usually attributed to selective release or founder events related to restricted gene flow in island settings. Here we document rapid morphological changes in rodents in 20 of 28 museum series collected on four continents, including 15 of 23 mainland sites. Approximately 17,000 measurements were taken of 1302 rodents. Trends included both increases and decreases in the 15 morphological traits measured, but slightly more trends were towards larger size. Generalized linear models indicated that changes in several of the individual morphological traits were associated with changes in human population density, current temperature gradients, and/or trends in temperature and precipitation. When we restricted these analyses to samples taken in the US (where data on human population trends were presumed to be more accurate), we found changes in two additional traits to be positively correlated with changes in human population density. Principle component analysis revealed general trends in cranial and external size, but these general trends were uncorrelated with climate or human population density. Our results indicate that over the last 100+ years, rapid morphological change in rodents has occurred quite frequently, and that these changes have taken place on the mainland as well as on islands. Our results also suggest that these changes may be driven, at least in part, by human population growth and climate change.

## Introduction

Humans are changing the global environment at unprecedented rates. Plants and animals can react to today's enormous environmental changes in one of three ways: they can move, they can adapt, or they can go extinct. Much attention has been focused on human-induced extinction, and some attention has been focused on movement of plants and animals in response to environmental change. However, relatively little research has addressed the ability of species to change either as a result of phenotypic plasticity or evolution in response to rapid environmental change.

Nonetheless, numerous instances of rapid morphological change have been documented. Most cases are thought to be caused either by pollution (e.g., industrial melanism in moths, heavy metal tolerance in plants) or by introductions of non-native organisms, usually with the introduced species itself evolving to meet the challenges of a new environment [1]. Changes in morphology and reproductive traits have been observed in a number of taxa, though these have been dominated by fishes (with changes resulting from fish stocking or selective fishing pressures) and birds [2]–[4].

In contrast to fishes and birds, rapid phenotypic change in mammals has been much more infrequently documented [1]–[2]. Although rapid change has been demonstrated in some other animals (e.g., selection, through hunting, for smaller bighorn sheep with smaller horns [5]), the great majority of changes have been in rodents on islands [6]. Such changes are usually attributed to selective release or founder events related to island settings [7]–[8], and are dependent on an island's size and its distance from the mainland [e.g. 6], [9]. However, recently Chicago-area white-footed mice also showed dramatic changes in morphology and mtDNA haplotype frequencies when invading urban environments [10]–[11]. Given complete genetic replacement, the morphological changes in these mice are best explained by population decline of one genotype and replacement (through migration) of another genotype better able to survive in local conditions. Movement or migration is a possible cause in some other cases of rapid morphological change, but most such cases do not have genetic components to help determine this.

Phenotypic plasticity, resulting from the behavioral and developmental responses of genotypes to environmental changes [12], may also cause rapid change. In a parallel example, high-altitude subspecies of deer mice were shown to genetically inherit higher oxygen-affinity hemoglobin, but these individuals also demonstrated phenotypic plasticity in the form of increased heart and lung size associated with increased oxygen consumption and increased gut size associated with energy uptake [13]. Maternal phenotypic effects (other than genetic effects) can also change fitness, and so alter phenotype frequencies [14]. Rapid selection of secondary sexual traits is also being documented more frequently, and usually involves changes in size, coloration, or courtship song [15].

There are a number of potential factors that could drive rapid morphological changes, whatever their mechanisms. Climate change has only recently begun to be implicated as a cause of rapid phenotypic change, but cases are now being documented with greater frequency [3], [15]. Microevolution for resistance to ozone pollution was documented in plantain [16], and rapid increase in growth rate in *Arabidopsis* in response to elevated atmospheric CO_2_ was demonstrated [17]. Rapid change in flowering time of turnips in response to variation in length in growing-season length has been documented [18]. Climate change has also caused rapid phenotypic change in butterflies [19] and birds [20]–[25]. A 47-year study of great tits in the UK showed a plastic shift in breeding date in response to climate [21]. Similarly, a study of red-billed gulls showed that while mean body mass increased with temperature there was no evidence of genetic change [25].

Urbanization is a second, major component of global change that has the potential to drive rapid morphological change. Obviously, increases in human population density are markers of increased anthropogenic effects of all kinds. For example, increasing human population density was hypothesized to cause loss of beak size bimodality in Darwin's finches [26]. Some anthropogenic effects, such as habitat loss and alteration, increase concurrently or near-concurrently with population density. Other anthropogenic effects, such as climate change, have substantially longer time lags.

To test the hypothesis that rapid morphological change is frequent in rodents on the mainland as well as on islands, and to investigate whether such change is being driven by either climate change specifically or increases in human populations generally, we sampled museum specimens of mammals collected over the last 100+ years. We measured 1302 specimens of 25 species in 28 museum series from 22 locations [Table 1], taking approximately 13,000 cranial measurements and recording some 4,000 external measurements. For each of the 28 series, we assessed local trends in climate and human population density over each of the collection periods. We then assessed whether and where change has occurred in rodent populations, and whether those changes were associated with either climate change or human population growth.

**Table 1:** Sample used in this study.

Case #	Family	Genus	Species	Subspecies	Continent	Country	State/Province	County/Dist (:Island)	<1950 N	.≥1950 N
1	Cricetidae	Abrothrix	longipilis	apta	SA	Chile	Los Lagos	Llanquihue	17	17
2	Cricetidae	Abrothrix	olivaceus	brachiotis	SA	Chile	Aisen	Aisen	26	25
3	Cricetidae	Abrothrix	olivaceus	brachiotis	SA	Chile	Los Lagos	Llanquihue	57	17
4	Cricetidae	Abrothrix	olivaceus	pencanus	SA	Chile	La Araucania	Malleco	17	30
5	Cricetidae	Abrothrix	sanborni		SA	Chile	Los Lagos	Chiloe	19	10
6	Cricetidae	Akodon	xanthorhinus	xanthorhinus	SA	Chile	Magallanes	Magallanes	54	23
7	Cricetidae	Lemmus	trimucronatus	nigripes	NA	USA	Alaska	Aleutians:St. George I.	29	23
8	Cricetidae	Microtus	mexicanus	mexicanus	SA	Mexico	Mexico	Toluca	20	18
9	Cricetidae	Microtus	pennsylvanicus	pennsylvanicus	NA	USA	Illinois	Cook	22	31
10	Cricetidae	Microtus	pennsylvanicus	pennsylvanicus	NA	USA	Wisconsin	Dodge	24	15
11	Cricetidae	Peromyscus	leucopus	noveboracensis	NA	USA	Illinois	Lake	54	45
12	Cricetidae	Peromyscus	maniculatus	anacapae	NA	USA	California	Ventura:Anacapa I.	39	58
13	Cricetidae	Peromyscus	maniculatus	blandus	NA	USA	New Mexico	Otero	16	14
14	Cricetidae	Peromyscus	maniculatus	elusus	NA	USA	California	Ventura:S. Barbara I.	29	23
15	Cricetidae	Peromyscus	maniculatus	nubiterrae	NA	USA	Kentucky	Harlan	18	18
16	Cricetidae	Peromyscus	maniculatus	santacruzae	NA	USA	California	Ventura:Santa Cruz I.	19	17
17	Geomyidae	Thomomys	umbrinus	umbrinus	SA	Mexico	Mexico	Toluca	13	14
18	Heteromyidae	Chaetodipus	fallax	fallax	NA	USA	California	San Diego	17	25
19	Heteromyidae	Dipodomys	merriami	merriami	NA	USA	Arizona	Yuma	36	38
20	Muridae	Lophuromys	flavopunctatus	zena	AFR	Kenya	Central	Kiambu	24	17
21	Muridae	Oligoryzomys	longicaudatus	philippii	SA	Chile	Aisen	Aisen	21	14
22	Muridae	Phyllotis	xanthopygus	chilensis	SA	Peru	Arequipa	Arequipa	12	12
23	Muridae	Phyllotis	xanthopygus	chilensis	SA	Peru	Arequipa	Caylloma	23	16
24	Muridae	Praomys	jacksoni		AFR	Kenya	Central	Kiambu	23	16
25	Muridae	Rattus	tanezumi	mindanensis	ASIA	Philippines	Negros I.	Negros Oriental	33	26
26	Sciuridae	Sciurus	carolinensis	pennsylvanicus	NA	USA	Illinois	Cook	13	19
27	Sciuridae	Tamias	striatus	griseus	NA	USA	Illinois	Lake	19	12
28	Spalacidae	Tachyoryctes	splendens	naivashae	AFR	Kenya	Rift Valley	Nakuru	10	8
								Totals	693	609

## Methods

### Specimen Selection

Our base criteria were that there should be a number of specimens collected both before and after 1950 in the same collection locality, defined as the county/district (or equivalent) level. This is the maximum level of geographic resolution likely to be encountered in museum specimens. We searched first for rodents from Families *Cricetidae* and *Muridae*, as most mammals in which rapid morphological change had been previously documented were from these families. We found 16 appropriate *Cricetidae* and six appropriate *Muridae* museum series. To increase the scope of the study, we added an additional six series consisting of *Heteromyidae*, *Sciuridae*, *Geomyidae*, and *Spalacidae*. These were the only additional series within *Rodentia* and meeting our criteria (collection before and after 1950, at least county/district locality resolution) that we found. Although the 25 resulting species are clearly not a random sample of rodents, they are all the species of rodents for which specimen coverage met these search criteria. We should note that a preponderance of *Cricetidae* and *Muridae* exists in museum collections overall: for example 67.5% of all *Rodentia* specimens at the National Museum are *Cricetidae* and *Muridae*. Data from Channel Island deer mice [6], [27]–[28] and Chicago-area white-footed mice [11] were included. By continent, 13 cases were from North America (US, including Alaska), 11 from South America (Chile, Mexico, and Peru), three from Africa (Kenya), and one from Asia (Philippines). There were 693 specimens collected before 1950, the earliest in 1892; and there were 609 specimens collected after 1950, the latest in 2001.

### Morphology

**Figure 1:**
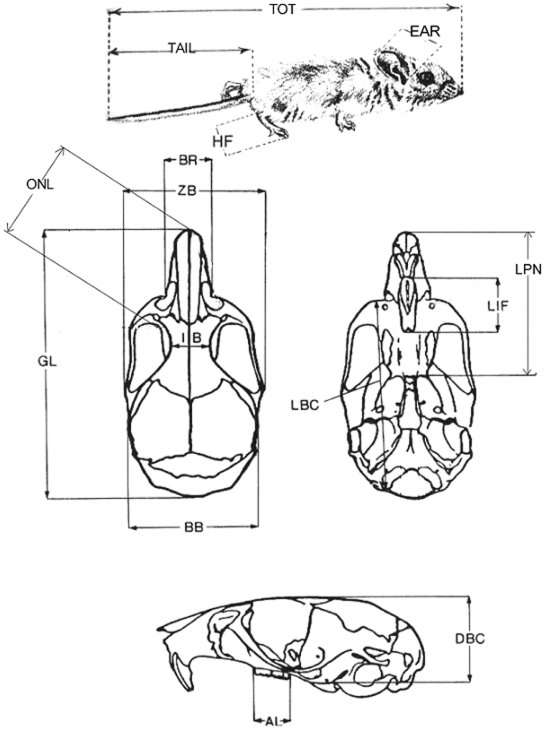
15 measurements used in this paper. Total length (TOT), tail length (TAIL), hind foot length (HF), ear length (EAR), alimentary toothrow (AL), breadth of braincase (BB), breadth of rostrum (BR), depth of braincase (DBC), greatest length of skull (GL), interorbital breadth (IB), length of braincase (LBC), length of incisive foramen (LIF), length of palate plus incisor (LPN, measured as the greatest distance from the end of the nasals to the mesopterygoid fossa), length from supraorbitals to nasals (ONL, measured as the least distance from the supraorbital notch to the tip of the nasals), and zygomatic breadth (ZB).

Eleven cranial measurements were taken following Collins and George [29], unless otherwise indicated. Measurements included: alimentary toothrow (AL), breadth of braincase (BB), breadth of rostrum (BR), depth of braincase (DBC), greatest length of skull (GL), interorbital breadth (IB), length of braincase (LBC), length of incisive foramen (LIF), length of palate plus incisor (LPN, measured as the greatest distance from the end of the nasals to the mesopterygoid fossa), length from supraorbitals to nasals (ONL, measured as the least distance from the supraorbital notch to the tip of the nasals), and zygomatic breadth (ZB) [Fig. 1]. All cranial measurements were taken by ORWP with a digital caliper, to the nearest 0.5 mm. The four standard external measurements in museum specimens were originally made by numerous different museum preparers and recorded from museum tags: total length (TOT), tail length (TAIL), hind foot length (HF), and ear length (EAR). Because of either lack of external measurement by museum preparers or damage to the skulls, some measurements were not available for some specimens. All measurements available to us were used. A spreadsheet containing all measurements may be found online at http://www.redrockinstitute.org/uploads/PergamsLawlerMeasurements.xls.

Normality of distribution was determined by visual inspection of normal probability plots [30] and Liliefors test [31]. SPSS v. 15.0 (SPSS, Inc. 2006) and SYSTAT v. 11.0 (SPSS, Inc. 2004) were used for statistical analyses.

To determine if morphological change had occurred, data were categorized into two time periods. We examined scatterplots of all collection years by case and determined that a cut point of the year 1950 was both appropriate to the data and logical in that it divided the collection range approximately equally. We performed independent-samples *t-*tests testing the significance of the difference between the sample means of the pre- and post-1950 time periods of each measurement of specimens at each location. Although we considered using regression analyses to examine trends in morphology over the entire collection period, the data were generally divided into two time periods, one in the earlier part of the 20^th^ century and one around the turn of the 21^st^ century. Thus, the data were more amenable to a categorical analysis. We used the Levene statistic [32] to test the assumption of equal variance, and applied the appropriate *t-*test. Though there have been recent and substantial objections to the use of sequential Bonferroni corrections [especially by ecologists; e.g. 33], to be conservative we then applied a Holm-Bonferroni sequential correction to account for the multiple tests being conducted [34]. We tabulated counts of significant increases and decreases in measurements as well as all changes (at Bonferroni adj. *p*<0.05) in each case.

Because we wished to evaluate rates of change as well as total amounts of change, we calculated the rate of annual change in each significant trait. This was done by dividing the difference in means by the difference in the means of collection years, both between periods. We displayed these data using box plots.

We also calculated rates of evolution in darwins with the equation 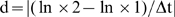

[35]

in which×1 and×2 are the mean value of each measurement in the time classes and Δt is time interval per million years.

To summarize changes in both size and shape over time, we performed principal component analyses (PCA) on the correlation matrix of the rates of changes of the 15 traits. The PCA was based on 28 replicates corresponding to the 28 series of specimens examined. Although all measurements used the same units (millimeters), there was an order of magnitude difference between external and cranial measures. Therefore, to avoiding having the external measures of body size dominate the PCA factors and obscure contributions of the skull morphology, we used the correlation matrix rather than the covariance matrix. Pairwise deletion was chosen to minimize losses of sample sizes because of missing character data. We chose the number of factors to retain by requiring minimum eigenvalues to equal 1.0, γ = 1.0000, and we used a Varimax orthogonal rotation. We used the first four factor scores as values for further analyses.

### Drivers of change

We explored the relationships between the changes in the morphological traits and potential environmental drivers based on changes in human population density and climate. Population data were estimated using data from the US Census Bureau and http://populstat.info. We calculated the trends in human population at each location between the mean of the year of the pre-1950 time period and the mean year of the post-1950 time period for each site [Table S1]. From the US Census we were able to estimate county-level population data for each US location from the closest decennial census. We were also able to obtain district/county-level data for Negros Island in the Philippines, but were able to obtain only province/state level data for most other locations. We were unable to get any population data for the early time-period for the Kenya locations.

Climate data were derived from the Climate Research Unit's TS2.1 global dataset [36]–[37]. The CRU dataset is a continuous global climatology for the period of 1901–2002 based on global meteorological station data modeled to half-degree resolution. We calculated trends in total annual precipitation and average annual minimum, maximum, and mean monthly temperatures as well as annual monthly minimum and maximum temperature and precipitation for the time period defined by the year of the first and last specimens sampled in each case. Trends were calculated as the slope of a linear regression of the given climate variable against time. Given the strong correlations among these variables, we chose to use trends in mean annual temperature and total annual precipitation in these analyses. Current temperatures were calculated as the mean annual temperature for the sample period at each site.

We explored potential associations between the general morphological changes and the four potential environmental drivers using general linear models. Although we expected some of these relationships to be non-linear, and perhaps modal, inspection of scatter plots provided no justification for non-linear models. We modeled each of four PCA factors as a function of trends in population density, current temperature, and trends in temperature and precipitation. We used general linear models with a backwards step-wise selection procedure with variable retention based on an α of 0.05. When appropriate, variables were transformed with power functions to meet assumptions of normality.

## Results

### Morphology

**Figure 2:**
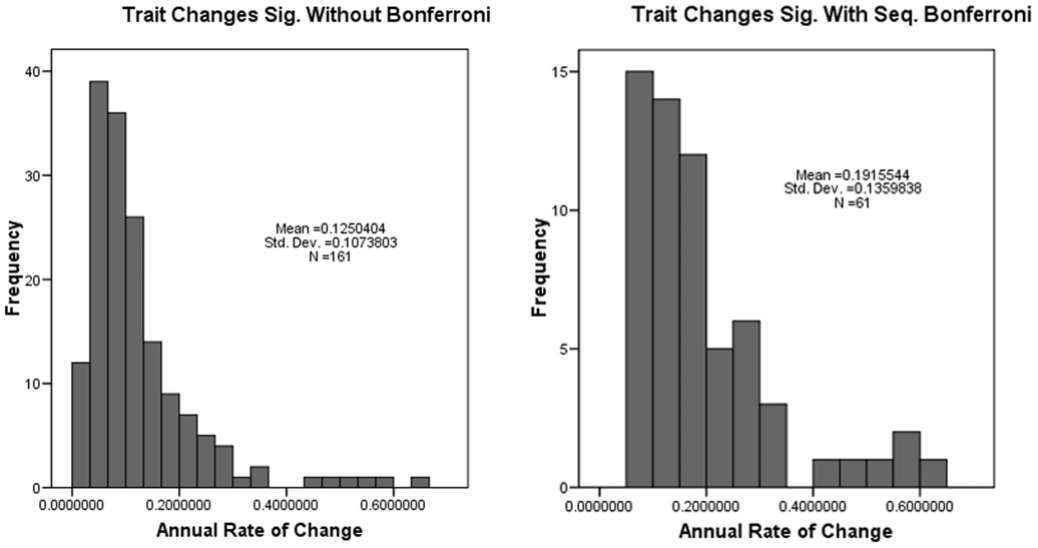
Histograms showing frequency distributions of significant trait changes before and after sequential Bonferroni correction. Left: frequency distribution of rates of annual changes significant before Bonferroni correction. Right: frequency distribution of rates of annual changes significant after sequential Bonferroni correction.

Of the 15 traits×28 series = 420 possible tests, 19 were not performed because measurements did not exist for at least one of the two time periods. This was usually because the external measure of ear length was not always taken pre-1950, but sometimes was because of cranial damage. Of the remaining 401 traits, 161 changed significantly at the 5% level before sequential Bonferroni correction. Using a corrected α of 0.05/401 = 0.000124 for the first test, 61 changes were still significant, or a mean of 2.2 changes per case [Table 2, Fig. 2]. There were 31 increases in measurements and 30 decreases. In general, significant changes were well distributed between external and cranial traits: there were 20 external changes and 41 cranial changes. The three cases with the greatest number of changes included two mainland sites and one island site and were very distant from each other: *Lophuromys flavopunctatus zena* (yellow-spotted brush-furred rat) from Kenya (7 changes), *Peromyscus maniculatus anacapae* (Anacapa Island deer mouse) from California (6 changes), and *Peromyscus leucopus noveboracensis* (northern white-footed mouse) from Illinois (5 changes).

**Table 2:** Table showing results of independent *t-*tests.

Case#	TOT	TAIL	HF	EAR	BR	ZB	ONL	GL	BB	IB	LBC	LIF	LPN	DBC	AL	POS	NEG	ABS	ABSMEAN
1								−0.0010			−0.0010				−0.0010		3	3	0.0010
2			−0.0006														1	1	0.0006
3																			
4																			
5																			
6																			
7	−0.0014	−0.0056	−0.0035	−0.0034													4	4	0.0035
8							0.0006									1			0.0006
9		−0.0028					0.0008									1	1	2	0.0018
10			−0.0018												−0.0008		2	2	0.0013
11				0.0016		0.0058		0.0047	0.0044		0.0050	0.0026				6		6	0.0040
12			−0.0015	0.0012	0.0007					−0.0020				−0.0006		2	3	5	0.0012
13						0.0012			0.0011							2		2	0.0011
14	−0.0018	−0.0023	−0.0019				0.0012									1	3	4	0.0018
15																			
16		−0.0027	−0.0016							−0.0026							3	3	0.0023
17																			
18				0.0064												1		1	0.0064
19				0.0017			0.0006			0.0006						3		3	0.0010
20	−−0.0015					−0.0013	−0.0013	−0.0011	−0.0009		−0.0011		−0.0011				7	7	0.0012
21								0.0007			0.0007					2		2	0.0007
22																			
23																			
24				0.0020	−0.0016					−0.0008					−0.0008	1	3	4	0.0013
25				0.0031								0.0020	0.0019	0.0029		4		4	0.0025
26														0.0025		1		1	0.0025
27											0.0009	0.0022				2		2	0.0015
28					0.0015		0.0020	0.0016					0.0016			4		4	0.0017
	3	4	6	7	3	3	6	5	3	4	5	3	3	3	3	31	30	61	

A sequential Bonferroni-Holm test was applied. Annual rates of change are the values given. NEG is the # traits that grew smaller over time, POS the # traits that grew larger, ABS the sum of the two, ABSMEAN the mean of absolute values.

**Figure 3:**
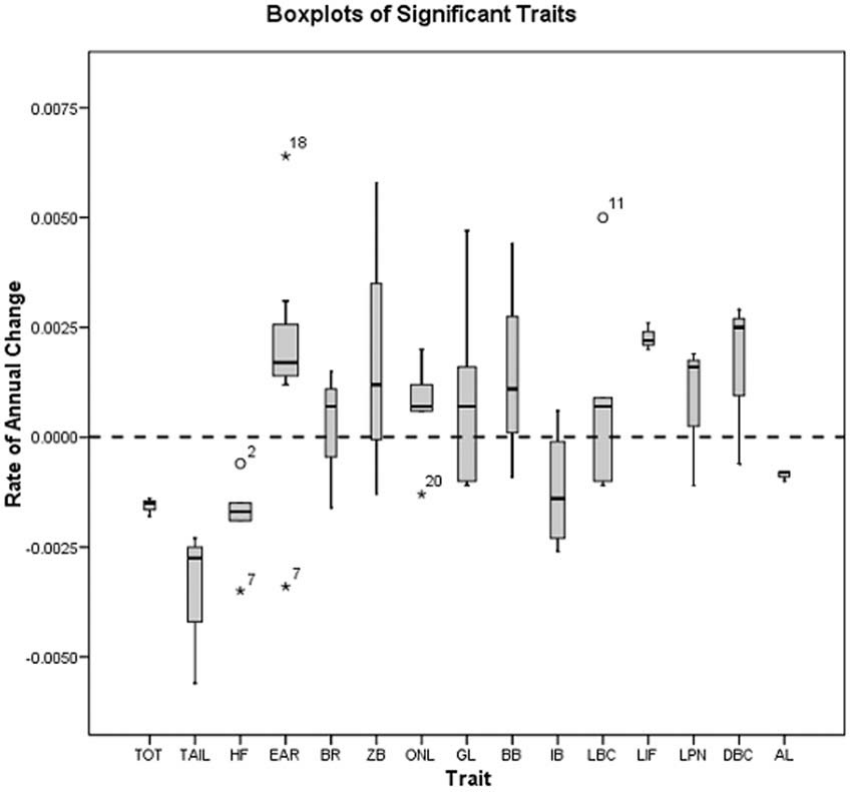
Box plots of rates of significant annual changes. Includes all cases for which changes were significant at p<0.05 after sequential Bonferroni correction. The length of the box is the interquartile range (IQR) computed from Tukey's hinges. Values more than three IQR's from the end of a box are extremes and are labeled with asterisks. Values more than 1.5 IQR's but less than 3 IQR's from the end of the box are outliers and are labeled with circles. Each extreme and outlier is labeled by case number as in Table 1.

Total percent change, annual percent change, and rate of change in darwins are given in Table S2. A graph of box plots of the rates of annual changes of significant traits is given in Fig. 3. This figure allows us to view the full range of significant changes for each trait, and how this range relates to a zero baseline. It also allows us to easily identify and gauge extreme and outlier cases. The greatest individual positive changes were 50.2% total over 79 years (0.63%/year, 6170 *d*) in ear length in *Chaetodipus fallax fallax* (northwestern San Diego pocket mouse) from California [Fig. 3, case #18] and 40.3% total over 69 years (0.58%/year, 5669 *d*) in zygomatic breadth in *Peromyscus leucopus noveboracensis* [Table S2]. The greatest negative changes were −27.0% total over 48 years (−0.56%/year, 5456 *d*) in tail length and −16.6% total over 48 years (−0.35%/year, 3353 *d*) in hind foot length, both in *Lemmus trimucronatus nigripes* (black-footed brown lemming) from Alaska's St. George Island [Fig. 3, case #7]. Rates of change of significant traits ranged from 549 to 6170 *d* (geometric mean = 1516 *d*), with 45 of the 61 traits changing at>1000 *d* [Table S2].

Principal component analysis explained of 73.9% of the variance with four factors [Table 3]. Factor I (explaining 38.6% of the variance) represents size of most cranial traits, with all 15 measures loading positively except ear length, which loaded negatively. Eight of these 15 measures have>0.5 loading. In contrast, Factor II (14.3%) consists primarily of external size measurements other than total length, and loading positively. Factor III (13.6%) discriminates using length of incisive foramen and depth of brain case. Factor IV (7.6%) has greatest contribution from total length.

**Table 3:** Rotated factor loadings, from a PCA on the correlation matrix.

Trait	Factor			
	1	2	3	4
TOT	0.04949	0.02104	0.0574	**0.9284**
TAIL	0.23423	**0.67735**	0.17732	−0.0455
HF	0.00167	**0.77485**	0.19238	0.03975
EAR	−0.037	**0.87023**	−0.0164	0.03834
BR	**0.76686**	0.16257	−0.3099	0.09852
ZB	**0.8343**	0.0176	0.34314	−0.2099
ONL	**0.8312**	−0.0598	0.29449	0.23954
GL	**0.9497**	0.0532	0.2245	0.08448
BB	**0.75366**	0.04721	0.1876	−0.1392
IB	0.1335	0.06827	0.75719	−0.0626
LBC	**0.89104**	0.04897	0.32303	0.03824
LIF	0.42365	0.19166	**0.53907**	0.28711
LPN	**0.8489**	0.07444	0.38397	0.10665
DBC	0.21083	0.33797	**0.61469**	0.11066
AL	**0.71308**	0.35233	−0.2722	0.16307
% σ^2^ exp.	38.546	14.267	13.505	7.618

We utilized pairwise deletion, the number of factors to retain by requiring minimum eigenvalues to equal 1.0, set γ = 1.0000, and performed a Varimax orthogonal rotation. Loadings>0.5 are in **bold**.

### Drivers of change

There were no clear associations between the general trends in morphology (as measured by the PCA factor scores) and the four potential environmental drivers. Given the lack of associations with the general trends, we explored relationships between the changes in individual traits and the four potential environmental drivers. We used generalized linear models with the same procedure described above for the models built for the PCA scores. Because the US population data were obtained with consistent standards, were always available to at least county-level resolution, and were gathered by a single source (US Census Bureau), we had substantially higher confidence in these data. Accordingly, in addition to building models for all cases, we also built separate models for the US cases. Sequential Bonferroni correction was also applied to these 15 traits×4 potential drivers = 60 tests. Using a corrected α of 0.05/60 = 0.00083 for the first test, 15 relationships were found to be significant. Models built with the whole dataset revealed changes in five traits that were associated with the potential drivers [Table 4]. Changes in three traits (breadth of rostrum, length of palate plus incisor, and length from supraorbitals to nasals) were positively associated with changes in precipitation. Changes in two traits were associated with changes in temperature and a change in one trait (length from supraorbitals to nasals) was associated with a change in human population density. Models built with only the US cases revealed three associations with changes in human population density, three with changes in precipitation, one with the trend in temperature, and two with current temperature [Table 4].

**Table 4:** Summary of generalized linear models explaining the % difference in each of the listed morphological traits as a function of four potential environmental drivers.

	All Cases					US Cases				
	Percent deviance explained	Population density trend	Current temperature	Temperature trend	Precipitation trend	Percent deviance explained	Population density trend	Current temperature	Temperature trend	Precipitation trend
TOT						44	+			
TAIL	16			_						
HF						43	+			
EAR						40		+		
BR	19				+					
ONL	***58***	***+***			***+***	***75***	***+***		***–***	***+***
GL	16			+						
IB						47		–		
LPN	17				+	**55**				**+**
DBC						40				+

The “+” and “−”symbols denote variables that were included in the models with positive and negative parameter estimates, respectively. An explanation of the two to four letter codes representing the morphological traits can be found in the legend for Fig. 1. Models explaining>50% of deviance are in ***bold***.

## Discussion

Humans have the ability to greatly alter local and global environments. Species that can respond quickly to these changes through phenotypic plasticity, migration, or rapid evolution have a distinct advantage in a dynamic world dominated by rapid land-use and climatic change. Most (20) of the 28 cases in our study showed changes in at least one—and as many as 7—morphological traits over some portion of the last century. Thus, at least for the last 100 years, rapid morphological change in some rodents seems to be frequent, and occurs on the mainland as well as on islands. Many of the changes we highlight here represent substantial morphological alterations. To give some context, the rates of change we found range from 549 to 6170 *d* [geometric mean = 1516 *d*, Table S2], a range similar to those found in well-known examples of rapid evolution such as beak length in Florida soapberry bugs, wing length (but not beak length) in Galapagos finches, or female age (but not spot number or size) in Trinidadian guppies [2]. The rates of change in this study are larger but still on the order of those found in historical colonization events (about 500 *d*) but much lower than those measured in laboratory selection experiments [about 60,000 *d*; 38].

Although our results clearly demonstrate rapid morphological changes, they do not conclusively link these changes to particular environmental drivers. There were no relationships between the PCA factor scores summarizing the changes in morphological traits and the four potential environmental drivers. The fact that there were some associations between the changes in individual traits and each of the four drivers indicates that although trends in some morphological characteristics may be correlated, the drivers for the changes in those traits may well be different across cases. Indeed, some drivers may interfere with one another as well. This would explain why the PCA did not lead to any significant trends, as selection on size might be favored by some drivers and not by others.

The links between morphological changes and potential drivers that we did identify did not clearly support two established biogeographic rules explaining rates of morphological change. Using total length as a surrogate for body mass, we did not find significant relationships between total length and either current temperature or change in temperature over time. Accordingly, Bergmann's Rule [39] was not corroborated by our data. We did find that some individual traits changed significantly with either current temperature or change in temperature over time, but they changed in both directions. Greatest length of skull and ear length grew larger with increasing average annual current temperature, corroborating Allen's Rule [40]. However, tail length, intermeatus breadth, and occipital-nasal length grew smaller with increasing temperature, contradicting Allen's rule. A contributing factor to Allen's Rule may be that the growth of cartilage is partly dependent on temperature, which grows quicker at warm temperatures (41). Unfortunately this finding does not shed additional light on our results, because our two largely cartilaginous traits (ear and tail length) behaved in opposition when considering temperature. Evolutionary rates are generally expected to be faster in warmer climates [42]–[45]: and thus one could speculate that a positive association between ear length and temperature is due to the use of the ear by mammals for temperature regulation.

There were more significant associations when cases were restricted to those located in the US [even though statistical power was diminished, Table 4]. This may be because the US population data were more accurate or had greater geographic resolution. Most links were with changes in human population density over time and/or change in precipitation. Some of this makes intuitive sense: as human population density increases, so do quality and abundance of rodent food sources, and we might expect rodents to grow larger (total length and hind foot length). It also makes general sense that size is positively associated with precipitation. Precipitation is positively associated with net primary productivity—although the relationship also depends on solar radiation [46]—and thus more rain can lead to more vegetation and thus potentially more food resources. It is not clear, however, why such size increases should be restricted to two measures related to nasal length and a measure of braincase depth.

The trait with the most explained deviance was occipital-nasal length, a hybrid measure of nasal length as well as width [Fig. 1]. Using all cases globally, a model composed of changes in population density and precipitation explained 58% of deviance [Table 4]. Explanatory power went up even further using only US cases: 75% of deviance was explained with changes in population density, temperature, and precipitation. We might speculate that this trait increased with human population and precipitation because these led to greater food resources, but decreased with higher temperatures not because of endothermic reasons but because the trait might also be involved in olfaction and the search for food. Therefore the need for a large nasal cavity was less important and there was a decrease in size.

With additional studies, it might be possible to explore some of the hypotheses generated by our correlations between morphological changes and environmental factors. For example, by looking for morphological changes at sites that are known to have experienced each of four combinations of large temperature increases, no temperature increases, changes in food resources, and no changes in food resources, one could begin to tease apart the relationship of these two factors and occipital-nasal length. One could also use a combination of bioenergetic models and additional targeted sampling of specimens to explore the degree to which changes in ear-size could be a product of thermal regulation.

There are, of course, limitations to the conclusions we may draw from these data. Although we found some significant associations with potential environmental drivers, the majority of the changes in morphology were not linked to changes in climate or human population density. Although it may be that these factors have not played a major role in driving the morphological changes we measured, it is likely that we had insufficient sample sizes to detect all such linkages. There are also other potential drivers of these morphological changes that we did not measure (e.g., competition or predation pressure from an introduced species). It is also possible that some of the traits studied might be important in sexual selection, and not only natural selection.

Given the absence of genetic analyses, it is impossible for us to attribute the morphological changes we measured to evolution. Migration and gene flow can interact in complex ways, and can either promote or constrain adaptive divergence through either gene flow or demography [47]. Simply defined, evolution involves changes in the predominant genotypes of a local population as a result of natural selection [48]. The change is either a result of selection favoring a recent mutation or a previously rare genotype. Although it is possible that some or all of the phenotypic changes in our cases are the result of evolution, we do not claim that they have such origins. The museum skins in one case (*Peromyscus leucopus noveboracensis*) have been DNA sequenced, and morphological changes were found to be best explained by the decline of the local population and its replacement (through migration) with individuals with another genotype better able to survive in local conditions [10]–[11]. It is possible that at least some other cases in this study involve movement of populations. Determining whether the phenotypic changes we observed were the result of evolutionary change or migration would hypotheses would require sequencing the DNA of the museum skins from the other cases in which morphological changes were observed.

Alternately, we have noted phenotypic plasticity as another potential mechanism of rapid morphological change, and plasticity is notoriously difficult to distinguish from direct genetic evolution [3], [12], especially in historical samples. However, type and degree of phenotypic plasticity can also be adaptive, and can also be the result of natural selection [e.g., 49]–[50]. We should further remember that five of our 28 cases were on islands, and that rapid phenotypic change on islands is often related to gene flow, with founder effects and genetic drift permitting a great deal of stochasticity in the absence of direct selection. Specifically, the rate of microevolution in island rodents varies inversely with island size and directly with distance of the island to the nearest island or mainland [e.g., 6], though there is some evidence that this rate peaks on intermediate size islands and decreases on very large islands [9].

The conclusions that can be drawn from this study are also limited by the geography and taxonomic diversity of our sample. Of the 28 cases we sampled, only four were outside of North and South America: three from Africa (all in Kenya) and one from Asia (Philippines). We had to visit museums in person in order to find appropriate museum series, and funding and time restrictions prevented our doing so outside of the US. We feel confident that additional series exist, that an increased level of commitment would allow sampling of a globally much broader area. Also, we cannot generalize our results to apply to all rodent families: most (22/28) of our cases were *Cricetidae* and *Muridae*, with the remaining six cases split between *Heteromyidae*, *Sciuridae*, *Geomyidae*, and *Spalacidae*. So we have samples from only six of the 33 rodent families [51].

Despite these limitations, our results clearly demonstrate rapid morphological change in multiple rodent species from both island and mainland populations. Furthermore, some of these changes appear to be driven by altered climates and growing human populations. Species that are able to respond quickly to environmental changes, whether through phenotypic plasticity, movement, or evolution will have a higher probability of surviving the rapid human-driven land-use and climate changes projected for the coming centuries. Understanding which species and populations have the greatest potential for rapid morphological change, and how species and populations can be managed to enhance their potential to adapt (evolutionarily or otherwise) to rapid phenotypic change, will be critical for conserving biodiversity in the coming century.

## Supporting Information

Table S1 Changes in human population sizes.
(0.09 MB DOC)

Table S2 Total and yearly % change of traits, and darwins.
(0.16 MB DOC)

## References

[1] Pergams ORW, Kareiva P, Levin SA, Carpenter SR, Godfray HCJ, Kinzig AP, Loreau M (2009). Support services: A focus on genetic diversity.. The Princeton Guide to Ecology.

[2] Hendry AP, Kinnison MT (1999). The pace of modern life: Measuring rates of contemporary microevolution.. Evol.

[3] Gienapp P, Teplitsky C, Alho J, Mills J, Merilä J (2008). Climate change and evolution: disentangling environmental and genetic responses.. Mol Ecol.

[4] Hendry AP, Farrugia TJ, Kinnison MT (2008). Human influences on rates of phenotypic change in wild animal populations.. Mol Ecol.

[5] Coltman DW, O'Donoghue P, Jorgenson JT, Hogg JT, Strobeck C, Festa-Bianchet M (2003). Undesirable evolutionary consequences of trophy hunting.. Nature.

[6] Pergams ORW, Ashley MV (2001). Microevolution in island rodents.. Genetica.

[7] Dayan T, Simberloff D (1998). Size patterns among competitors: ecological character displacement and character release in mammals, with special reference to island populations.. Mammal Rev.

[8] Palkovacs EP (2003). Explaining adaptive shifts in body size on islands: a life history approach.. Oikos.

[9] Heaney L (1978). Island area and body size of insular mammals: Evidence from the tri-colored squirrel (*Calloscuirus prevosti*) of Southeast Asia.. Evol.

[10] Pergams ORW, Barnes WM, Nyberg D (2003). Rapid change of mouse mitochondrial DNA.. Nature.

[11] Pergams ORW, Lacy RC (2007). Rapid morphological and genetic change in Chicago-area *Peromyscus*.. Mol Ecol.

[12] Ghalambor CK, McKay JK, Carroll SP, Reznick DN (2007). Adaptive versus non-adaptive phenotypic plasticity and the potential for contemporary adaptation in new environments.. Func Ecol.

[13] Hammond KA, Roth J, Janes DN, Dohm MR (1999). Morphological and physiological responses to altitude in deer mice (*Peromyscus maniculatus*).. Phys Biochem Zool.

[14] Räsänen K, Kruuk LEB (2007). Maternal effects and evolution on ecological timescales.. Func Ecol.

[15] Svensson E, Gosden T (2007). Contemporary evolution of secondary sexual traits in the wild.. Func Ecol.

[16] Davison AW, Reiling K (1995). A rapid change in ozone resistance of *Plantago major* after summers with high ozone concentrations.. New Phyt.

[17] Franks SJ, Sim S, Weis AE (2007). Rapid evolution of flowering time by an annual plant in response to a climatic fluctuation.. Proc Nat Acad Sci USA.

[18] Ward DE, Kengen SWM, van der Oost J, de Vos WM (2000). Purification and characterization of the alanine aminotransferase from hyperthermophilic archaeon *Pyrococcus furiosus* and its role in alanine production.. J Bacteriol.

[19] Zakharov EV, Hellmann JJ (2009). Genetic differentiation across a latitudinal gradient in two co-occurring butterfly species: revealing population differences in a context of climate change.. Mol Ecol. In press.

[20] Both CJ, Sanz J, Artemyev AA, Blaauw B, Cowie RJ (2006). Pied flycatchers traveling from Africa to breed in Europe: differential effects of winter and migration conditions on breeding date.. Ardea.

[21] Charmantier A, McCleery RH, Cole LR, Perrins C, Kruuk LEB (2008). Adaptive phenotypic plasticity in response to climate change in a wild bird population.. Science.

[22] Möller AP, Szep T (2005). Rapid evolutionary change in a secondary sexual character linked to climatic change.. J Evol Biol.

[23] Garant D, Sheldon BC, Gustafsson L (2004). Climatic and temporal effects on the expression of secondary sexual characters: genetic and environmental components.. Evol.

[24] Hegyi G, Torok J, Toth L, Garamszegi LZ, Rosivall B (2006). Rapid temporal change in the expression and age-related information content of a sexually selected trait.. J Evol Biol.

[25] Teplitsky CJ, Mills A, Alho JS, Yarrall JW, Merilä J (2008). Bergmann's rule and climate change revisited: Disentangling environmental and genetic responses in a wild bird population.. Proc Nat Acad Sci USA.

[26] Hendry AP, Grant PR, Grant BR, Ford HA, Brewer MJ (2006). Possible human impacts on adaptive radiation: beak size bimodality in Darwin's finches.. Proc Roy Soc B.

[27] Pergams ORW, Ashley MV (1999). Rapid morphological change in Channel Island deer mice.. Evol.

[28] Pergams ORW, Ashley MV, Browne DR, Mitchell KL, Chaney HW (2000). California Island deer mice: genetics, morphometrics, and evolution.. Proceedings of the Fifth California Islands Symposium: 29 March to 1 April 1999.

[29] Collins PW, George SB (1990). Systematics and taxonomy of island and mainland populations of western harvest mice (*Reithrodontomys megalotis*) in southern California.. Contributions in Science, Nat Hist Mus Los Angeles Cty.

[30] Afifi AV, Clark A, May S (2004). Computer-Aided Multivariate Analysis, 4th ed..

[31] Lilliefors H (1967). On the Kolmogorov-Smirnov test for normality with mean and variance unknown.. J Amer Stat Assoc.

[32] Brown MB, Forsythe AB (1974). Robust tests for the equality of variances.. J Amer Stat Assoc.

[33] Moran MD (2003). Arguments for rejecting the sequential Bonferroni in ecological studies.. Oikos.

[34] Holm S (1979). A simple sequentially rejective multiple test procedure.. Scand J Stat.

[35] Haldane JBS (1949). Suggestions as to quantitative measurement of rates of evolution.. Evol.

[36] Mitchell TD, Carter TR, Jones PD, Hulme M, New M (2004). A comprehensive set of high-resolution grids of monthly climate for Europe and the globe: the observed record (1901–2000) and 16 scenarios (2001–2100).. Tyndall Centre Climate Chge Res Work Paper 55.

[37] Mitchell TD, Jones PD (2005). An improved method of constructing a database of monthly climate observations and associated high-resolution grids.. Intl J Climatology.

[38] Gingerich P (1983). Rates of evolution: effects of time and temporal scaling.. Science.

[39] Bergmann C (1847). Über die Verhältnisse der Wärmeökonomie der Thiere zu ihrer Grösse.. Gottinger Studien.

[40] Allen JA (1877). The influence of Physical conditions in the genesis of species.. Radical Rev.

[41] Serrat MA, King D, Lovejoy CO (2008). Temperature regulates limb length in homeotherms by directly modulating cartilage growth.. Proc Nat Acad Sci USA.

[42] Janzen DH (1967). Why mountain passes are higher in the tropics.. Amer Nat.

[43] Rohde K (1992). Latitudinal gradients in species diversity: The search for the primary cause.. Oikos.

[44] Wright S, Keeling J, Gillman L (2006). The road from Santa Rosalia: A faster tempo of evolution in tropical climates.. Proc Nat Acad Sci USA.

[45] Kozak KH, Wiens JJ (2007). Climatic zonation drives latitudinal variation in speciation mechanisms.. Proc Roy Soc B: Biol Sci.

[46] Rosenzweig ML (1968). Net primary productivity of terrestrial communities: prediction from climatological data.. Amer Nat.

[47] Garant D, Ford SE, Hendry AP (2007). The multifarious effects of dispersal and gene flow on contemporary adaptation.. Func Ecol.

[48] Hartl DL, Clark AG (2007). Principles of Population Genetics, 4th ed..

[49] Via S, Gomulkiewiczb R, De Jongc G, Scheinerd SM, Schlichting CD (1995). Adaptive phenotypic plasticity: consensus and controversy.. Trends Ecol Evol.

[50] Agrawal AA (2001). Phenotypic plasticity in the interactions and evolution of species.. Science.

[51] Wilson DE, Reeder DM (2005). Mammal Species of the World: A Taxonomic and Geographic Reference, 3rd ed..

