# Supplementary material for: Recent and Widespread Rapid Morphological Change in Rodents
Source: PLoS One. 2009 Jul 31;4(7):e6452. doi: 10.1371/journal.pone.0006452 (PMC2714069; doi:10.1371/journal.pone.0006452)
Supplement: Table S1 — Changes in human population sizes. (0.09 MB DOC) [file pone.0006452.s001.doc]

Table S1. Changes in human population sizes. “Pop Early” refers to the approximate human population at the mean year of the sample collected before 1950 (“Year Mean Early”), “Pop Late” refers to the approximate human population at the mean year of the sample collected after 1950 (“Year Mean Late”), and “Pop Diff” refers to the change in human population size between these mean years. County/District -level data is used for US data and within Negros Island, State/Province level data is used otherwise.

| Case# | Country | State/Province | County/District (: Island) | Area (m^2^) | Pop Early | Pop Late | Pop Diff |
| --- | --- | --- | --- | --- | --- | --- | --- |
| 1, 3 | Chile | Los Lagos | Llanquihue | 7107 | 113700 | 252316 | 138616 |
| 2 | Chile | Aisen | Aisen | 33396 | 3963 | 71668 | 67705 |
| 4 | Chile | La Araucania | Malleco | 5512 | 119938 | 203208 | 83270 |
| 5 | Chile | Los Lagos | Chiloe | 9053 | 113650 | 120647 | 6997 |
| 6 | Chile | Magallanes | Magallanes | 52285 | 35450 | 112726 | 77276 |
| 7 | USA | Alaska | Aleutians: St. George I. | 35 | 0 | 163 | 163 |
| 8 | Mexico | Mexico | Toluca | 8286 | 798500 | 2478490 | 1679990 |
| 9 | USA | Illinois | Cook | 1635 | 3053017 | 5240904 | 2187887 |
| 10 | USA | Wisconsin | Dodge | 907 | 45681 | 75064 | 29383 |
| 11 | USA | Illinois | Lake | 1368 | 104387 | 644356 | 539969 |
| 12 | USA | California | Ventura: Anacapa I. | 1 | 20 | 10 | -10 |
| 13 | USA | New Mexico | Otero | 6627 | 6689 | 44665 | 37976 |
| 14 | USA | California | Ventura: Santa Barbara I. | 1 | 0 | 0 | 0 |
| 15 | USA | Kentucky | Harlan | 468 | 75275 | 41889 | -33386 |
| 16 | USA | California | Ventura: Santa Cruz I. | 250 | 100 | 100 | 0 |
| 17 | Mexico | Mexico | Toluca | 8286 | 809275 | 2478490 | 1669215 |
| 18 | USA | California | San Diego | 4526 | 34987 | 1357854 | 1322867 |
| 19 | USA | Arizona | Yuma | 5519 | 7733 | 60827 | 53094 |
| 20 | Kenya | Central | Kiambu | 5087 |  | 1706625 |  |
| 21 | Chile | Aisen | Aisen | 33396 | 3963 | 70489 | 66526 |
| 22, 23 | Peru | Arequipa | Arequipa, Caylloma | 24528 | 277846 | 608267 | 330421 |
| 24 | Kenya | Central | Kiambu | 5087 |  |  |  |
| 25 | Phillipines | Negros Oriental | Negros I. | 13327 | 1453650 | 3095560 | 1641910 |
| 26 | USA | Illinois | Cook | 1635 | 4063342 | 5403908 | 1340566 |
| 27 | USA | Illinois | Lake | 1368 | 74285 | 516418 | 442133 |
| 28 | Kenya | Rift Valley | Nakuru | 67131 |  | 4807480 |  |
