# Supplementary material for: Recent and Widespread Rapid Morphological Change in Rodents
Source: PLoS One. 2009 Jul 31;4(7):e6452. doi: 10.1371/journal.pone.0006452 (PMC2714069; doi:10.1371/journal.pone.0006452)
Supplement: Table S2 — Total and yearly % change of traits, and darwins. (0.16 MB DOC) [file pone.0006452.s002.doc]

Supplementary Table 2. Total and yearly % change of traits, as well as rate of change expressed in darwins.

| Case | Trait | Mean Year  Early Period | Mean Year  Late Period | Difference  Years | Total %  Change | % Change/  Year | darwins |
| --- | --- | --- | --- | --- | --- | --- | --- |
| 18 | EAR | 1894 | 1973 | 79 | 0.50232 | 0.00636 | 6170 |
| 11 | ZB | 1926 | 1995 | 69 | 0.40311 | 0.00584 | 5669 |
| 7 | TAIL | 1917 | 1965 | 48 | -0.26988 | -0.00562 | 5456 |
| 11 | LBC | 1926 | 1995 | 69 | 0.34268 | 0.00497 | 4819 |
| 11 | GL | 1926 | 1995 | 69 | 0.32507 | 0.00471 | 4572 |
| 11 | BB | 1926 | 1995 | 69 | 0.30005 | 0.00435 | 4220 |
| 7 | HF | 1917 | 1965 | 48 | -0.16585 | -0.00346 | 3353 |
| 7 | EAR | 1917 | 1965 | 48 | -0.16317 | -0.00340 | 3299 |
| 25 | EAR | 1948 | 1988 | 40 | 0.12258 | 0.00306 | 2974 |
| 25 | DBC | 1948 | 1988 | 40 | 0.11568 | 0.00289 | 2806 |
| 9 | TAIL | 1922 | 1995 | 73 | -0.20273 | -0.00278 | 2695 |
| 16 | TAIL | 1933 | 1971 | 38 | -0.10253 | -0.00270 | 2618 |
| 11 | LIF | 1926 | 1995 | 69 | 0.18239 | 0.00264 | 2565 |
| 16 | IB | 1933 | 1971 | 38 | -0.09706 | -0.00255 | 2479 |
| 26 | DBC | 1941 | 2001 | 60 | 0.15207 | 0.00253 | 2460 |
| 14 | TAIL | 1932 | 1975 | 43 | -0.09925 | -0.00231 | 2240 |
| 27 | LIF | 1920 | 1992 | 72 | 0.15652 | 0.00217 | 2110 |
| 24 | EAR | 1906 | 1956 | 50 | 0.10129 | 0.00203 | 1966 |
| 25 | LIF | 1948 | 1988 | 40 | 0.08061 | 0.00202 | 1955 |
| 28 | ONL | 1906 | 1988 | 82 | 0.16456 | 0.00201 | 1947 |
| 12 | IB | 1940 | 1980 | 40 | -0.07960 | -0.00199 | 1931 |
| 14 | HF | 1932 | 1975 | 43 | -0.08305 | -0.00193 | 1874 |
| 25 | LPN | 1948 | 1988 | 40 | 0.07553 | 0.00189 | 1832 |
| 14 | TOT | 1932 | 1975 | 43 | -0.07570 | -0.00176 | 1708 |
| 10 | HF | 1903 | 1977 | 74 | -0.13004 | -0.00176 | 1705 |
| 19 | EAR | 1909 | 1974 | 65 | 0.11231 | 0.00173 | 1677 |
| 11 | EAR | 1926 | 1995 | 69 | 0.11035 | 0.00160 | 1552 |
| 16 | HF | 1933 | 1971 | 38 | -0.06041 | -0.00159 | 1543 |
| 28 | LPN | 1906 | 1988 | 82 | 0.13012 | 0.00159 | 1540 |
| 28 | GL | 1906 | 1988 | 82 | 0.12890 | 0.00157 | 1525 |
| 24 | BR | 1906 | 1956 | 50 | -0.07854 | -0.00157 | 1524 |
| 12 | HF | 1940 | 1980 | 40 | -0.06032 | -0.00151 | 1463 |
| 20 | TOT | 1906 | 1964 | 58 | -0.08591 | -0.00148 | 1437 |
| 28 | BR | 1906 | 1988 | 82 | 0.11967 | 0.00146 | 1416 |
| 7 | TOT | 1917 | 1965 | 48 | -0.06777 | -0.00141 | 1370 |
| 20 | ZB | 1906 | 1964 | 58 | -0.07604 | -0.00131 | 1272 |
| 20 | ONL | 1906 | 1964 | 58 | -0.07552 | -0.00130 | 1263 |
| 13 | ZB | 1934 | 1982 | 48 | 0.05902 | 0.00123 | 1193 |
| 12 | EAR | 1940 | 1980 | 40 | 0.04824 | 0.00121 | 1170 |
| 14 | ONL | 1932 | 1975 | 43 | 0.04945 | 0.00115 | 1116 |
| 20 | GL | 1906 | 1964 | 58 | -0.06582 | -0.00113 | 1101 |
| 20 | LPN | 1906 | 1964 | 58 | -0.06273 | -0.00108 | 1050 |
| 20 | LBC | 1906 | 1964 | 58 | -0.06240 | -0.00108 | 1044 |
| 13 | BB | 1934 | 1982 | 48 | 0.05105 | 0.00106 | 1032 |
| 1 | GL | 1939 | 1983 | 44 | -0.04566 | -0.00104 | 1007 |
| 1 | LBC | 1939 | 1983 | 44 | -0.04436 | -0.00101 | 978 |
| 1 | AL | 1939 | 1983 | 44 | -0.04296 | -0.00098 | 948 |
| 27 | LBC | 1920 | 1992 | 72 | 0.06655 | 0.00092 | 897 |
| 20 | BB | 1906 | 1964 | 58 | -0.04921 | -0.00085 | 823 |
| 24 | IB | 1906 | 1956 | 50 | -0.04224 | -0.00084 | 820 |
| 9 | ONL | 1922 | 1995 | 73 | 0.05973 | 0.00082 | 794 |
| 10 | AL | 1903 | 1977 | 74 | -0.05839 | -0.00079 | 766 |
| 24 | AL | 1906 | 1956 | 50 | -0.03745 | -0.00075 | 727 |
| 21 | GL | 1923 | 1987 | 64 | 0.04605 | 0.00072 | 698 |
| 21 | LBC | 1923 | 1987 | 64 | 0.04416 | 0.00069 | 670 |
| 12 | BR | 1940 | 1980 | 40 | 0.02655 | 0.00066 | 644 |
| 12 | DBC | 1940 | 1980 | 40 | -0.02476 | -0.00062 | 601 |
| 8 | ONL | 1892 | 1963 | 71 | 0.04291 | 0.00060 | 586 |
| 19 | IB | 1909 | 1974 | 65 | 0.03927 | 0.00060 | 586 |
| 2 | HF | 1923 | 1986 | 63 | -0.03623 | -0.00058 | 558 |
| 19 | ONL | 1909 | 1974 | 65 | 0.03678 | 0.00057 | 549 |
